# StOP? II trial: cluster randomized clinical trial to test the implementation of a toolbox for structured communication in the operating room—study protocol

**DOI:** 10.1186/s13063-022-06775-y

**Published:** 2022-10-18

**Authors:** Sandra Keller, Franziska Tschan, Norbert K. Semmer, Sven Trelle, Tanja Manser, Guido Beldi

**Affiliations:** 1 Department of Visceral Surgery and Medicine, Inselspital, Bern University Hospital, University of Bern, Bern, Switzerland; 2 Institute for Work and Organizational Psychology, University of Neuchâtel, Neuchâtel, Switzerland; 3 Department of Psychology, University of Bern, Bern, Switzerland; 4 CTU Bern, University of Bern, Bern, Switzerland; 5 FHNW School of Applied Psychology, University of Applied Sciences and Arts Northwestern Switzerland, Olten, Switzerland

**Keywords:** Operating room, Teamwork, Communication, Coordination, Briefing, Checklist, Post-operative complications, Mortality, Cluster randomized controlled trial

## Abstract

**Background:**

Surgical care, which is performed by intensely interacting multidisciplinary teams of surgeons, anesthetists, and nurses, remains associated with significant morbidity and mortality. Intraoperative communication has been shown to be associated with surgical outcomes, but tools ensuring efficient intraoperative communication are lacking. In a previous study, we developed the StOP?-protocol that fosters structured intraoperative communication. Before the critical phases of the operation, the responsible surgeon initiates and leads one or several StOP?s. During a StOP?, the surgeon informs about the progress of the operation (status), next steps and proximal goals (objectives), and possible problems (problems) and encourages all team members to voice their observations and ask questions (?). In a before-after study performed mainly in visceral surgery, we found effects of the StOP?-protocol on mortality, length of hospital stay, and reoperation. We intend to assess the impact of the StOP?-protocol in a cluster randomized trial, in a wider variety of surgical specialties (i.e., general, visceral, thoracic, vascular surgery, surgical urology, and gynecology). The primary hypothesis is that the consistent use of the StOP?-protocol by the main surgeon reduces patient mortality within 30 days after the operation. The secondary hypothesis is that the consistent use of the StOP?-protocol by the main surgeon reduces unplanned reoperations, length of hospital stay, and unplanned hospital readmissions.

**Methods:**

This study is designed as a multicenter, cluster-randomized parallel-group trial. Board-certified surgeons of participating clinical departments will be randomized 1:1 to the StOP? intervention group or to the standard of care (control) group. The intervention group will undergo a training to use the StOP?-protocol and receive regular feedback on their compliance with the protocol. The surgeons in the control group will communicate as usual during their operations. The unit of observation will be operations performed by cluster surgeons. Consecutive patients will be enrolled over 4 months per cluster. A total of 400 surgeons will be recruited, and we expect to collect patient outcome data for 14,000 surgical procedures.

**Discussion:**

The StOP?-protocol was designed as a tool to structure communication during surgical procedures. Testing its effects on patient outcomes will contribute to implementing evidenced-based interventions to reduce surgical complications.

**Trial registration:**

ClinicalTrials.gov NCT05356962. Registered on May 2, 2022

**Supplementary Information:**

The online version contains supplementary material available at 10.1186/s13063-022-06775-y.

## Background

Each year, about 4.2 million people die within 30 days following a surgical procedure [1]. Further decreasing mortality after surgical procedures therefore warrants continuing effort. Surgical quality has an impact not only on mortality but also on other clinical outcomes such as unplanned reoperations [2].

Outcomes of operations depend on many factors. Patient factors are pivotal; additional factors include structural as well as procedural aspects [3]. Regarding the quality of the surgical procedure proper, surgeon experience and surgeon caseload [4, 5] as well as surgeon technical skills [6, 7] play a major role.

There is mounting evidence, however, that communication and teamwork within the surgical team are important for patient outcomes, and studies are needed that aim at improving teamwork and assessing clinical patient outcomes [8]. Research showed problems in about 30% of communication during operations, causing delays, tensions in the team, inefficiencies and procedural errors [9], undesirable incidents [10], or flow disruptions, that in turn may lead to procedural errors [11]. Regarding *patient outcomes*, our own observational study [12] demonstrated a significantly lower risk of surgical site infections (SSI) when the surgical team communicated about task-related aspects more frequently. In the same study, we observed a higher risk of SSI for teams showing more case-irrelevant communication at the end of the operation, corroborating the results of another study [13] showing a significant relationship between *intraoperative* information sharing and patient outcomes.

Core aspects of effective communication are creating and maintaining high levels of situation awareness in surgical teams—this is a (shared) perception and knowledge regarding important elements and the development of the situation that indicate (or allow to predict) a need for action [14, 15]. Situation awareness requires a shared understanding of the characteristics and requirements of the task in general (often called “shared mental model”) [14]. Another interesting focus is on communication practices that aim at team reflexivity [16, 17], which includes not only an overt reflection in the team, but also reflective communication about the team’s objectives, strategies, and processes [18]. In-action team reflection (reflecting during the process) was related to team performance in simulated medical emergencies [17].

Common methods to improve communication in the OR are based on *checklists* and briefings [19], typically conducted before or after an operation. Checklists help to structure the communication of clinically relevant information and were introduced because missing or faulty information regarding important aspects of the operations (e.g., patient identity, surgical procedure) represented a serious threat to patient safety. However, if checklists are too long, people may develop “checklist fatigue” [20], regard them as trivial or obvious, and may reduce adherence or use it as a “checkbox exercise” [21]. A second approach to improve communication is based on *general teamwork and communication training*, typically conducted in a specific training setting. Although such training settings may be realistic (e.g., high-fidelity simulations) [22], they can only teach general principles (e.g., “you should make sure that your communication has been received properly”) that need to be transferred to other situations. However, transfer of training is not easy to achieve [23], and training effects may not sustain over time [24]. Furthermore, it is often not easy to motivate participants for training. In surgery, this applies notably for experienced surgeons [25]. Success, i.e., reduction in intraoperative problems, morbidity, and mortality, has been reported both for the introduction of checklists [21] and for teamwork training [26–28]. However, findings are not unequivocal for both [8, 21, 28, 29]. Difficulties and obstacles to transferring training to clinical practice are regularly reported in the literature [21, 25, 30]. Careful implementation of interventions into surgical practice thus requires particular attention.

### The StOP?-protocol

In an attempt to encourage structured information exchanges during surgical procedures, we developed an intervention aimed to assure structured information exchange during operations, the StOP?-protocol [31]. The StOP?-protocol is a short intra-operative briefing, initiated by the main surgeon with the aim to inform the entire OR team about the operation. First, the surgeon describes the current *St*atus of the operation (i.e., what has been done, what they are currently doing), then states the *O*bjectives (i.e., the next steps of the operation), the potential *P*roblems they may encounter, and, finally, asks if the team members have contributions or questions (?). The briefing is very short (30–90 s) and requires the attention of the entire OR team (including anesthesia and circulators), who is asked to stop working during the briefing. The StOP?s are used in a flexible way at times when they do not interrupt the flow of the operation or actions that require full attention of the surgeon. The surgeons thus choose when they perform the StOP?-protocol and are asked to announce during the pre-operative team timeout when they intend to perform the StOP?-protocol(s).

In a before-and-after design, the StOP?-protocols were introduced in four surgical departments of four different hospitals (mostly general surgery) in 8256 operations [31] (herein referred to as the StOP? I study). Adjusted intention-to-treat analyses did not show an effect of the StOP?-protocol for surgical site infections (SSI) but did reveal a reduced risk for mortality, unplanned reoperations, and length of stay. The per-protocol analysis supported these findings. Despite promising results, the study has limitations inherent to before-after studies, and sample sizes were calculated based on rates of SSI. To generalize the results, the study needs to be replicated with more hospitals and a more advanced study design. These considerations led us to test the effects of the StOP?-protocol as part of a randomized controlled trial, in a large number of hospitals and various surgical specialties.

## Methods/design

### Objectives

The main objective of the trial is to test if performing the StOP?-protocol improves patient outcomes after operations. The underlying rationale is that by influencing clinical communication within the surgical team during the operation, the StOP?-protocol may improve the quality and safety of surgical care and thereby reduce post-operative mortality and morbidity.

The primary hypothesis is that the consistent use of the StOP?-protocol by the main surgeon reduces patient mortality within 30 days after an operation.

The secondary hypothesis is that the consistent use of the StOP?-protocol by the main surgeon reduces unplanned reoperations and unplanned readmissions up to 30 days after the index operation, and reduces the length of hospital stay.

### Study design

**Fig. 1:**
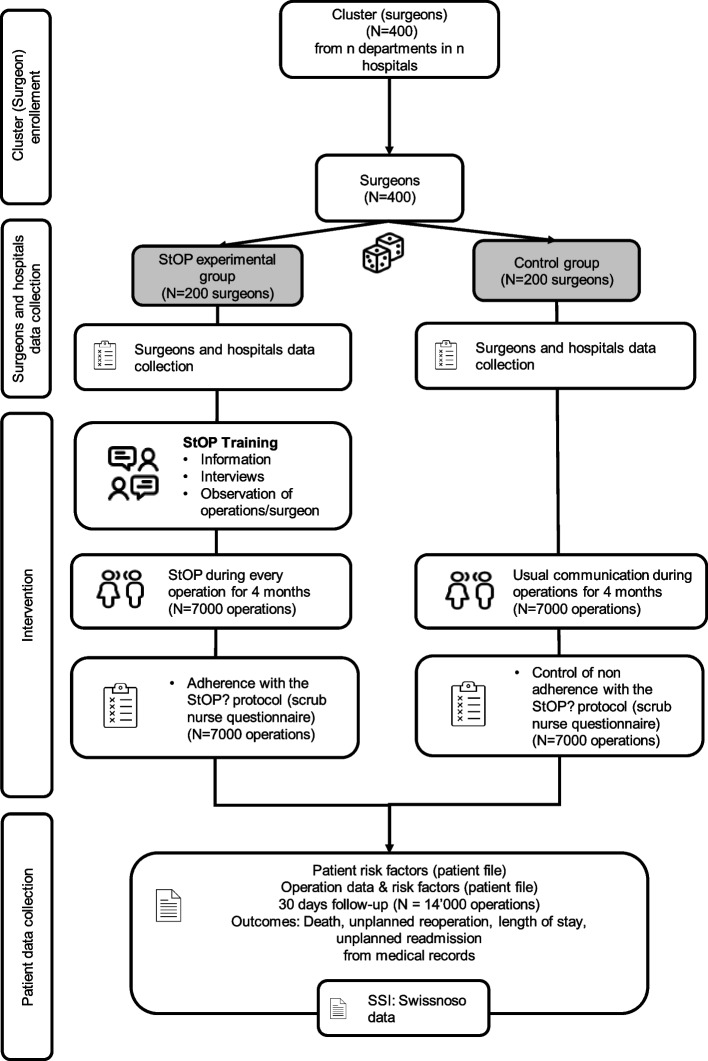
Study design

This study is designed as a multicenter, cluster-randomized parallel-group trial. Board-certified surgeons of participating clinical departments form the cluster units will be randomized 1:1 to the StOP? intervention group or to the standard of care group. The unit of observation will be operations performed by cluster surgeons. Consecutive patients will be enrolled over approximately 4 months per cluster (see Fig. 1 for an overview).

We expect that a behavioral intervention targeting the clinical communication of the surgeon will impact the behavior of the entire team present. Surgeons in the intervention group will be trained in the StOP?-protocol with the aim of performing this intervention routinely and consistently during their operations. Because surgeons are specifically trained to adaptively perform the StOP?-protocol, a trial randomizing individual patients or operations is not a feasible study design.

The individual surgeon represents a cluster instead of larger units such as departments or hospitals, for the following reasons: First, the main surgeons have the deepest knowledge of the procedure and its course; furthermore, they bear responsibility for the outcome. Their communication behavior therefore is central to the surgical process [13]. Second, in most hospitals, surgical team stability is rather low. Constant retraining of team members would be necessary if an intervention was dependent on all team members being trained. Taking the individual surgeon as a cluster allows to train the core person to perform the intervention independently of the actual team composition.

### Setting and participants

Study sites will be invited to participate in this trial based on the availability of eligible clusters and operations. The inclusion and exclusion criteria are defined for the cluster level (i.e., the surgeons) and the operation level.

#### Study sites

Study sites, i.e., departments employing surgeons specialized in general, visceral, thoracic, vascular surgery; surgical urology; or gynecology, will be eligible to participate in the study. This choice was guided by the mortality rates of about 1% in these specialties [32–39], which is comparable to general and abdominal surgery included in the StOP? I. Traumatology departments or general surgical departments that include traumatology are included. Cardiac surgery was excluded because of the higher incidence of mortality and already established communicative behaviors typically between the surgeon and the perfusionist. Neurosurgery was excluded because of the minimal number of high-risk procedures performed by a very limited number of surgeons.

#### Surgeons

Board-certified surgeons in one of the specialties of interest will be recruited. Surgeons who are already performing StOP?-protocols (e.g., because they took part in the first StOP? study and surgeons who were previously enrolled in the StOP? II trial (e.g., because they moved or rotated between participating sites) will be excluded. Resident surgeons will not be recruited as clusters, since they either assist or perform surgical procedures under supervision, and since they are still in training, an additional task may interfere with their technical performance.

#### Operations

Elective and emergency operations performed by a cluster (surgeon) during the cluster-specific time period will be considered for inclusion. We expect an operation volume of 30 eligible operations per surgeon within 4 months. This number is the target per cluster but the actual number will vary among surgeons.

Exclusion criteria for operations include the following:

1. Patient age below 18 years
2. Previous operation at the same site up to 30 days prior to the index operation
3. Procedures not done in operating rooms but in outpatient clinics, onwards, etc.
4. Mainly diagnostic endoscopic procedures (e.g., colonoscopy, gastroscopy, bronchoscopy)
5. Percutaneous interventions (e.g., transurethral interventions)
6. Documented refusal of the patient for the use of healthcare-related data

### Recruitment, screening, and informed consent procedure

#### Surgeons

On the participating study sites, board-certificated surgeons will receive detailed information on the study to decide on participating. Each participating surgeon will sign an informed consent defining tasks, responsibilities, potential risks, and benefits. The investigators will explain to each surgeon the nature of the study, its purpose, the procedures involved, and the expected duration. Each surgeon will be informed that the participation in the study is voluntary and that he or she may withdraw from the study at any time.

On the consent form, participants will be asked if they agree to the use of their data should they choose to withdraw from the trial. Participants will also be asked for permission for the research team to share relevant data with people from the universities taking part in the research or from regulatory authorities, where relevant.

#### Patient consent

The StOP?-protocol is an intraoperative briefing addressing communication within the surgical team. The study has no impact on the medical treatment of the patient. Therefore, this study is performed based on a general consent from the patients, where they agree with the use of healthcare-related data that are the result of the patient’s treatment.

### Intervention

The surgeons in the intervention group will undergo a multi-module training on how to use the StOP?-protocol [31]. The training will encompass the following steps: (1) Documentation: the surgeons in the intervention group will receive documentation on the StOP?-protocol, including a leaflet describing the StOPs? and when and how to use them during operations. (2) In-person instructional interview: in a one-to-one instruction, training instructors will conduct a training interview with each surgeon in the intervention group (planned duration: 20 min). Goals of the interviews are to identify the most appropriate moments to initiate StOP?s for the surgical procedures the surgeon performs most often, to discuss details on how to announce and perform the protocol, and to discuss open questions. (3) On-site feedback for at least three operations led by surgeons in the interventional group in the first 2 weeks of the intervention period. An instructor will be present in the OR and observe how the StOP?-protocol is announced and performed. A short, in-person feedback will be given immediately after the operation. (4) During the whole intervention period, adherence to the StOP?-protocol will be assessed at least monthly and results communicated to each surgeon; in the case of adherence issues, the research team will offer support to discuss potential barriers to adherence.

Surgeons in the control group will not be trained. They represent the standard of care, using local practices, and are asked to communicate as usual during their operations. Given the nature of the study, these surgeons will not be blinded to the intervention.

In each participating site, the anesthesiology and OR nurses team will be informed about the study. The information will consist of a presentation of the study by the research team and flyers will be available to inform about the StOP?-protocol and the study goals.

### Measurements

The following data will be collected.

#### Hospital and surgical units

Basic characteristics of the study sites will be recorded, such as the language region, hospital type, number of beds, number of employees, number of operating rooms, yearly operating volume, hospital teaching category, number of staff in training, current practices of the WHO timeout and signouts, and previous team training history.

### Participating surgeons

Data collected about all participating surgeons include age, gender, nationality, native language, year of primary medical degree, year of general and specialized surgery degrees, tenure at the current hospital, tenure in current position; hierarchy level, job title, study arm, and time frame of participation in the study. For surgeons in the intervention group, additional information collected are adherence rates and whether the initial training, and retrainings took place.

#### Patient and operation characteristics

We will collect data about the type of operation, date of admission, date of operation, time of incision, time of last stich, duration, surgical access, level of urgency, and IDs of the surgeons and residents present during the operation. Patient characteristics include gender, age at the time of operation, height, weight, previous laparotomies at the same site 30 days prior the operation, ASA score, and wound contamination class.

#### Intervention adherence

The intervention is a behavioral intervention that requires the surgeon to remember and implement a new behavior pattern; thus, adherence needs to be assessed. Adherence to the StOP?-protocol will be measured for each operation. Adherence is assessed using a standardized reporting form filled in by each scrub technician present during the operation before they leave the operating room. They report if and how many StOP?s were performed during their presence and provide a simple quality rating of the StOP?s on a Likert scale from 1 (very bad) to 5 (very good); a definition of the characteristics of a good StOP? is provided (i.e., spoken loudly enough, complete (StOP? acronym), engaged, the complete team stopped working and paid attention). Adherence of reporting by the scrub technicians will be calculated by comparing the number of operations performed with the number of reports submitted. Adherence to the StOP?-protocol will be calculated by comparing the adherence reports of the scrub technicians for surgeons in the intervention group with the number of operations performed by these surgeons.

#### Endpoints

The primary endpoint is death up to 30 days after the start of the index operation. This information will be provided by the hospital. The hospital obtains the information from patient records and, if not available, via the patients directly, the family, or the general practitioner.

The following secondary endpoints will be assessed:

1. Unplanned reoperation within 30 days is defined as any operation after the end of the index operation for up to 30 days. Only reoperations within the same hospital of the index operation will be considered. The definition of type IIIb (surgical reoperation) of the Clavien-Dindo Classification will be used to define this variable [40]. Data will be obtained from medical records.
2. Length of index hospital stay is defined as days between the index operation to discharge or transfer to another hospital. In-hospital deaths will be recorded and considered as censoring events. Data will be obtained from medical records.
3. Unplanned readmission within 30 days is defined as any unplanned readmission at the same hospital as the index hospital within 30 days after the operation. Readmissions that are mentioned already in the discharge letter of the index hospital stay are considered planned if the reason for the readmission matches to the one described in the discharge letter. Data will be obtained from medical records.
4. We plan to conduct additional analyses on surgical site infections as an endpoint. Data on surgical site infections are collected according to international standards defined by the Centers for Disease Control and Prevention (CDC) by the surveillance system of Swissnoso—the national center for the prevention of infections in Switzerland. Type 1 (superficial), type 2 (fascial), and type 3 (organ/space) infections are distinguished (91). Swissnoso collects data only in Switzerland and only for a subset of operations. The data will be obtained from Swissnoso and form a pragmatic subsample of data that is available.

### Withdrawal and discontinuation

Based on the previous study (StOP? I) [31], retention problems with regard to surgeons and patients are likely to be very low. This assumption is justified by the relatively short enrollment period per surgeon and the short follow-up for the primary endpoint. Even if patients drop out, ascertaining whether patients are alive at 30 days is often still possible. With regard to patient retention, the biggest risk is that patients do not give or withdraw consent to use their data which we deem unlikely given the low patient burden.

### Randomization

#### Allocation sequence

Enrolled clusters will be randomized in a 1:1 fashion to either the StOP? or the standard arm. Randomization will be stratified by surgical department and seniority (Chef-/Leitende Ärztin/Arzt versus other).

#### Concealment mechanism

Allocation will be done using a web-based system, which also contains the electronic case report form (eCRF): REDCap. The system reveals the allocation only after a cluster is definitely enrolled in the trial, i.e., eligibility criteria confirmed.

#### Implementation

Dedicated study personnel will establish a list of eligible surgeons. After obtaining informed consent from the surgeons, the responsible study personnel will transfer the list of enrolled clusters to CTU Bern. There, an independent data manager will allocate these clusters to the two trial groups (batch randomization) and will create a new cluster record within the web-based system. The system will disclose the assignment only after the completion of this registration.

### Statistical analysis plan and sample size calculation

#### Sample size calculation

Sample size calculations were based on the primary objective, i.e., whether patients in the StOP? intervention group have a lower risk of mortality compared to patients in the standard of care group. The primary effect measure will be odds ratio as the primary analysis will be based on a logistic regression (see below). Based on the data from the first StOP? study, we calculated an intracluster correlation coefficient (ICC) of 0.024 from a mixed-effects logistic model. Also, we estimated 30-day mortality from the before phase of the StOP? study and use this number (1.8%) for the event rate in the control group. The target treatment effect that we do not want to miss is an odds ratio of 0.5, i.e., we aim to reduce mortality in the StOP? intervention group to 0.9%. Sample size calculations were done in Stata (release 16.1) using the power twoproportions command with the cluster option. The command calculates power based on a *z*-test with standard error adjusted by the intracluster correlation and varying cluster size. With 400 clusters overall (with 1:1 allocation, i.e., 2 × 200), an average size of 24 patients per cluster, and a coefficient of variation of 0.5, we will achieve 84% power at a significance level of 0.05 (two-sided).

We assume that some patients will be operated by two disparate surgeons (i.e., two surgeons randomized to different trial arms are performing one operation jointly). To accommodate this, we fixed the target average cluster size at 30 patients. This sample size allows for varying cluster sizes to accommodate differences in operation volume across clusters (coefficient of variation of 0.5, i.e., cluster size is allowed to range between 10 and 50 patients). A minimum of 12,000 patients will be required. Based on the experience of the StOP? I study, we expect that some surgeons perform many more operations than the assumed upper cluster size. Therefore, we expect that 14,000 patients will be enrolled overall. Given the relatively low number of events, we performed simulation studies to check the robustness of the assumption of an asymptotically normally distributed test statistic. Using a random-effects logistic regression in 1000 simulated trials with the same assumptions as described above, we calculated a power of more than 90%.

We also performed several sensitivity analyses to account for the uncertainty of our assumptions, e.g., (1) allowing for a larger variation in cluster size (coefficient of variation of 0.7) results in 82% power; (2) allowing for the unlikely case of higher intracluster correlation (ICC of 0.03) results in 80% power; and (3) allowing for enrolment problems i.e., 370 clusters but similar cluster size results in 80.9% power or a similar number of clusters but smaller average cluster size (21 patients) results in 80.6% power.

#### Statistical method for primary and secondary outcomes

The primary analysis will follow the intention-to-treat (ITT) principle. We will assess outcomes at the patient level, accounting for the correlated nature of data within surgeon and hospital by using multilevel mixed-effects models. The primary outcome (30-day mortality) will be presented as a number and proportion for each group. We will incorporate random intercepts for surgeons and units in the multilevel models and adjust for variables related to the site, surgeon characteristics, and type of operation. The odds ratio of death for the intervention vs. the control group will be presented with a two-tailed 95% confidence interval (CI) and accompanying *p*-value. The primary null hypothesis of no difference in mortality between the two groups will be rejected if the two-tailed *p*-value is < 0.05.

Binary secondary outcomes (unplanned reoperation and readmission) will be analyzed with the same model approach as used for the primary outcome. For the time-to-event outcome length of stay, we will use a multilevel mixed-effects parametric survival model parameterized as an accelerated failure time model. Random and fixed factors will be used as described above. The results will be presented as the difference in days with 95% CIs. Length of stay will be censored at the time of death or transfer to another hospital. Depending on the number of in-hospital deaths, we will consider a competing risk regression as the primary analysis approach for this outcome. Differences between the groups will be evaluated using multilevel mixed-effects linear regression, adjusting for random and fixed factors as described above. The results will be presented as the mean difference with 95% CI.

We will report results in accordance with the 2010 Consolidated Standards of Reporting Trials (CONSORT) statement extension to cluster-randomized trials [41].

#### Statistical methods for additional analyses

As a secondary analysis, we will perform a per-protocol analysis, excluding operations where the surgeon in the intervention group did not adhere to the protocol and where surgeons in the control group actually performed a StOP?. However, it is now well known that a naïve per-protocol analysis is problematic as it does not properly take confounding/selection bias into account [42]. Direct and indirect adjustment methods are described to estimate the per-protocol effect of a point intervention such as the StOP?-protocol [43]. We will explore the possibility to directly adjust for confounders by using inverse probability weighting [43].

Because cluster randomization may lack the optimal balancing in characteristics between the groups seen in individual-level randomization, we will adjust each model for additional pre-defined patient-level, surgeon-level, and hospital-level variables to account for case-mix differences between the groups in a sensitivity analysis. Moreover, we will qualitatively assess the imbalances of hospital, surgeon, and patient characteristics between the groups. If we observe relevant imbalances in covariates not considered in the primary and sensitivity analyses, we will perform additional adjustments and consider propensity score-based approaches to assess the robustness of our results.

In exploratory analyses, we will assess the adherence and study the influence of the number of StOP?-protocols on outcomes. Such analyses include the assessment of the impact of surgeons’ gender or patients’ gender on primary or secondary endpoints.

We plan exploratory analyses based on the collected data. One such analysis concerns predictors of adherence to the protocol, based on adherence data. We also plan to investigate the changes in outcomes based on surgeon experience with the StOP?-protocol (number of operations with StOP?. Further exploratory analyses will assess the impact of surgeon and patient gender on adherence and on endpoints.

*Interaction between the treatment and the control group*: An important aspect is that surgeons in the control group may be operating together with surgeons in the intervention group within a surgical department. Three different combinations are expected: (i) both surgeons are from the intervention group (25% of interactions), (ii) both surgeons are from the control group (25% of interactions), and (iii) one surgeon is from the intervention group and the other from the control group (50% of interactions). The observations from the StOP? I study showed that collaborations between board-certified surgeons are much more common in university (90% of the procedures) and large teaching (30% of procedures) hospitals, and less frequent in rural hospitals (5% of the procedures). We take this into account for the sample size calculation and the data analyses.

We will conduct exploratory analyses for operations jointly performed by surgeons that are assigned to different treatment groups with a special emphasis on the influence of hierarchical status on adherence to the StOP?-protocol.

#### Analysis of population and missing data

The main analysis will be based on the intention-to-treat analysis set where all operations of a randomized surgeon done during the enrolment period are analyzed in the randomized arm, regardless of adherence. The per-protocol analysis set consists of all operations where the surgeon was adherent, i.e., if a surgeon in the control group performs a StOP?-protocol during an operation, such operations will be excluded and, if a surgeon in the intervention group does not perform a StOP?-protocol, such operations will be excluded.

We do expect only few missing data with respect to the primary outcome (30-day mortality). We will impute missing outcome data based on all available baseline characteristics of hospitals, surgeons, and patients using chained equations. Analyses will be conducted as described above using Rubin’s rules to combine measures across multiple data sets [44]. This will be done for the analyses of the primary endpoint and the three key secondary endpoints.

#### Reproducibility

We will deposit the final protocol in a preprint repository (medRxiv.org) and submit it to a peer-reviewed journal. At publication of the primary results, the full protocol, the data management plan including the codebook of the trial database, and information on how to get access to the primary trial data will be published in the repository of the University of Bern which fulfills the FAIR criteria (Bern Open Repository and Information System (boris.unibe.ch)). A 12-month grace period after publication of the primary results is currently foreseen to allow all collaborators to complete additional, pre-planned analyses and projects.

#### Interim analyses

We will conduct one interim analysis after endpoint data were completely collected for 200 clusters. We will reassess the ICC based on the data collected to adjust the sample size if needed. To calculate the ICC, we will use multilevel mixed-effects logistic regression. If the ICC is > 0.024, we will perform new power calculations using the same assumptions as in the original sample size calculation. Based on these calculations, we will increase the number of clusters and/or the number of patients per cluster except if the required increase would be too large to be feasible. Because the type I error is not affected by the recalculation of the ICC, we will not adjust the significance level.

Moreover, an interim analysis of the primary outcome will be performed to examine the trial for potential futility: The conditional power for a statistically significant difference between the intervention and control groups at the end of the study will be estimated based on data collected at the time of the interim analysis. If the conditional power is < 20%, it is recommended to terminate the trial for futility. Implications for type I and II errors are minimal, so no adjustment is needed for this type of interim analysis [45].

Given that no interim analysis for safety or efficacy will be done, no independent Data Monitoring Board (iDMC) is established, and possible sample size adaptations or trial termination for futility will be decided by the Steering Committee.

#### Blinding

This is an open-label trial as the intervention cannot be blinded. Given the nature of the primary endpoint, the risk for ascertainment bias is considered negligible. Follow-up data collection is centralized and will be done by blinded personnel.

#### Schedule

The schedule and timelines are presented in Tables 1 and 2.

**Table 1:** Timeline related to the clusters wit hin one participating unit

Timeline Cluster (Surgeon)									
Time points are related to start of StOP? implementation per cluster	before intervention	up to -4 week to start of intervention	up to 2 weeks to intervention	first 2 weeks of intervention	during intervention, for each operation	1 mo after start of intervention	2 mo after start of intervention	3 mo after start of intervention	4 mo after start of intervention
**All surgeons **									
Surgeon recruitment	X								
Eligibility screening	X								
Informed consent	X								
Allocation to intervention or control group		X							
Demographic data collection		X							
Hospital and surgical unit characteristics	X	X							
Measuring adherence with StOP? Protocol / if StOP?s were performed (questionnaires filled out by the scrub nurses)					X				
**Surgeons in the intervention group**									
StOP? training and on-site feedback			X	X					
Feedback and refresher training						X	X	X	
End of intervention report									X

**Table 2:** Timeline related to the collection of operation and patient-related data

Timeline of aquisition of patient and operation related data	During intervention, for each operation	End of every week of intervention	1 month after start of intervention	2 month after start of intervention	3 month after start of intervention	4 month after start of intervention	5+ month after start of intervention
**Measuring adherence**							
Measuring adherence with StOP? Protocol (questionnaires filled out by the scrub nurses)	X						
Basic characteristics of operations		X					
Generation of patient key		X					
**Measuring patient characteristics and outcomes**							
Patient and operation characteristics and outcome data			X	X	X	X	X

#### Study schedule

**Fig. 2:**
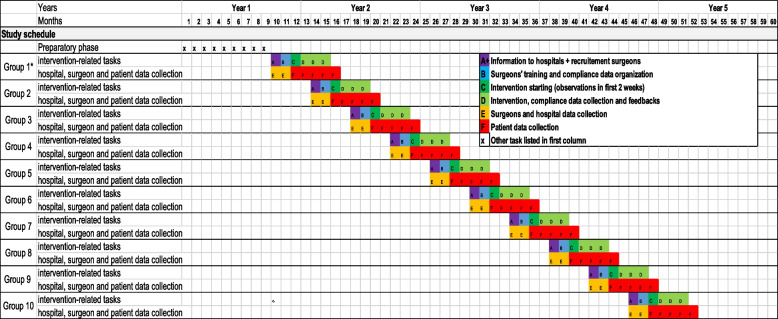
Timeline for the participating surgical units

Figure 2 shows the timeline related to the participating units. Note that units are grouped according to their geographic location, because the intervention requires a high level of presence of the researchers in participating units. The study is performed in 40 centers that include university and tertiary centers in Switzerland, Germany, and Austria. Recruitment of centers is ongoing. Currently, letters of commitment are available from 28 centers.

### Regulatory aspects and safety

#### Local regulations/Declaration of Helsinki

This study is conducted in compliance with the protocol, the current version of the Declaration of Helsinki, ICH-GCP in analogy, and applicable national regulations, i.e., the Human Research Act in Switzerland as well as other locally relevant legal and regulatory requirements.

#### (Serious) adverse events and notification of safety and protective measures

StOP? II is regulated by chapter 4 of the Clinical Trial Ordinance. Chapter 4 describes the safety documentation and notification requirements for serious adverse events (SAEs). It defines (Art. 63) SAEs that have to be documented (and reported) as any event which:

- Requires inpatient treatment not envisaged in the protocol or extends a current hospital stay
- Results in permanent or significant incapacity or disability
- Is life-threatening or results in death
- Causes a congenital anomaly or birth defect

And it cannot be excluded that the event is attributable to the intervention.

Given the nature of the intervention, we submit that Article 63 is not directly applicable to this trial. Unless there are specific reasons to assume an involvement of the StOP?-protocol, we will not routinely record serious adverse events and therefore also not foresee this in the case report forms and standard operating procedures. We base this decision on the following reasons: (1) the intervention is not directly applied to patients; (2) the nature of the intervention and the perceived mechanism practically exclude any direct effects; rather, the intervention entails its effects (if any) indirectly (see Methods/design); consequently, we can a priori exclude any relationship between the intervention and the events listed above; (3) even establishing a temporal relationship of any untoward medical occurrences to the intervention is difficult to operationalize; (4) finally, we fear that the heterogeneity of the underlying operations and the patient population would result in a high number of recorded events (if any event is documented) and actually blur any safety issues.

Instead, we record relevant data with regard to safety as primary and secondary endpoints. 

#### Reporting of serious adverse events

Not applicable as described above

#### Follow-up of serious adverse events

Not applicable as described above

#### Notification of safety and protective measures

If immediate safety and protective measures have to be taken during the conduct of the study, the investigator notifies the responsible ethics committee of these measures, and of the circumstances necessitating them, within 7 days.

#### (Periodic) safety reporting

An annual safety report (ASR/DSUR) containing relevant safety information from all sites is provided by the sponsor. Relevant safety information in this case means descriptive statistics of accumulating primary and secondary endpoint events pooled across trial arms. Pooling of trial arms is necessary to avoid multiple testing. The sponsor distributes the ASR/DSUR to all the participating Investigators. It is submitted once a year to the local ethics committee by the investigator.

#### Amendments

Substantial changes to the study setup and study organization, the protocol, and relevant study documents are submitted to the Ethics Committee for approval before implementation. Under emergency circumstances, deviations from the protocol to protect the rights, safety, and well-being of human subjects may proceed without prior approval of the Ethics Committee. Such deviations shall be documented and reported to the Ethics Committee as soon as possible.

Substantial amendments are changes that affect the safety, health, rights, and obligations of participants, changes in the protocol that affect the study objective(s) or central research topic, changes of study site(s), or of study leader and sponsor.

A list of substantial changes is also available on www.swissethics.ch.

A list of all non-substantial amendments will be submitted once a year to the responsible Ethics Committee together with the ASR.

#### (Premature) termination of study

The sponsor may terminate the study prematurely according to certain circumstances, e.g.:

- ○ Ethical concerns
- ○ Insufficient participant recruitment
- ○ Alterations in accepted clinical practice that make the continuation of the study unwise
- ○ Early evidence of harm of the experimental intervention

Upon regular study termination, the Ethics Committee is notified within 90 days.

Upon premature study termination or study interruption, the Ethics Committee is notified within 15 days.

#### Insurance

To compensate trial participants for study-related damage or injuries, liability is provided per local regulations.

#### Confidentiality and coding

Trial and participant data will be handled with utmost discretion and is only accessible to authorized personnel who require the data to fulfill their duties within the scope of the study. On the CRFs and other study-specific documents, participants are only identified by a unique participant number.

The following data will be centrally stored:

1. Characteristics of participating hospitals (hospital data)
2. Characteristics of participating clinical departments (department data)
3. Characteristics of participating surgeons including allocated trial arm and training details (surgeon data)
4. Patient key
5. Characteristics, operation, and outcome data of the patient (patient data)
6. Adherence to the StOP?-protocol

Linkage of data will be ensured via unique keys. Data 1–4 will be stored in a GCP-compliant trial management system on the same server. Data 1, 2, and 4 do not contain directly identifying person data. The surgeon data (#3) contains the name of the surgeon. This will be mentioned explicitly in the informed consent. The identifying data of the surgeon will only be visible for data entry personnel. The system prohibits to generate lists of names or to export the names. Given the data that must be collected for each surgeon and the limited number of surgeons per clinical department, we consider it identifying whether or not it contains the name.

Identifiable data of patients will be stored in a physically separated GCP-compliant database. Access to the database will be restricted to data entry personnel. After the closure of the database containing participant data, each site will receive a list of their patients (participant log) for archiving. After all sites confirmed receipt of their list, the database will be (completely) erased. A codebook describing the set-up of the database will be stored in the Trial Master File. The data on adherence to the StOP?-protocol will be collected via questionnaires filled out by the scrub technicians after each operation (#6). To identify the operation, the scrub technicians will put the patient sticker on the paper questionnaire and deposit the questionnaire in a sealed box within the operation room ward. The paper questionnaires will at no point leave the hospital. The research team will enter the questionnaires into the system, and the data will be coded at this point, so that no identifying information will remain with the electronically stored adherence data.

#### Retention and destruction of study data and biological material

All study data are archived for at least 15 years after study termination or premature termination of the study.

### Further aspects

#### Overall ethical considerations

The expected results may reveal if clinical outcomes can be improved by addressing team behavior during the performance of complex surgical procedures. In particular, the study has the potential to provide evidence that a short (typically between 30 and 90 s) and inexpensive intraoperative briefing may reduce mortality and morbidity. The results of the study will be of interest not only to surgical specialists but also to a wide public, and therefore, progression and findings will be communicated to healthcare workers of other specialties.

#### Risk-benefit assessment

As benefits, we expect that this simple, cost-efficient behavioral intervention will improve the quality of peri-operative communication and consequently reduce the risk of errors associated with the intervention and peri-operative care, eventually reducing post-operative patient mortality, unplanned reoperations, readmissions, and reduce the length of hospital stay. If the study reveals a positive effect not only on mortality, it is likely that the StOP?-protocol has importance for even more types of operations including operations of the head and neck and orthopedic procedures, and it may be extended to disciplines that mainly perform percutaneous interventions such as cardiology, angiology, and neuroradiology that typically are associated with other complications than mortality.

From a clinical perspective, the risks of the study seem minimal. In the StOP? I trial, we did not observe adverse events or risks associated with the intervention itself. The primary treatment is not affected at any time by these interventions. Distraction or irritation of the personnel or prolongation of the surgical procedures because of the briefings has not been observed between the two groups. Surgeons in the intervention group perform the StOP?-protocol according to the manuals and training. We will not define the criteria for modifying the intervention, as the surgeon is allowed to adapt the timing and execution of the protocol according to the procedure. This flexibility of the StOP?-protocol is a central part of the concept. Overall risks of StOP? seem comparable to the risk of conducting preoperative time-out that is now standard worldwide.

However, from a scientific perspective, there are some risks inherent to this study:

*Threat of bias of contamination*: Given that the study aims to change the behavior of surgeons in the treatment group, potential bias or contamination of the surgeons enrolled in the control group needs to be addressed:

- ○ First, surgeons in the control group will not undergo the StOP? training, but they will not be blind to the intervention as surgeons within a department work closely together. This could be a source of contamination, because surgeons in the comparator group may observe StOP?s performed while they operate with a surgeon in the intervention group. If surgeons in the comparator group observe communication behavior that they judge as promising, they may be motivated to copy this behavior. However, sporadic observations of others’ behaviors are not sufficient to sustainably change a behavior. Active strategies are needed to improve adherence to and ensure the sustainability of behavioral interventions, such as audits and feedbacks [46].
- ○ Second, different hospitals will be included in the study and some may already have implemented a sign-out. Surgeons in the intervention group will explicitly be asked to reflect what extent the StOP?-protocol may be associated to the sign-outs, so that both can be combined during the operation in the case they already work with sign-outs. We will record the communication practices of each hospital (including sign-outs) and statistically control for these practices in the analysis.

*Threat of potential interaction between the treatment and the control group*: An important aspect is that surgeons in the comparator group may be operating together with surgeons in the treatment group within a surgical department. Thus, a not trained surgeon may participate in operations where a StOP?-protocol is done; this could lead to direct interactions. As discussed in the section on statistical methods for additional analyses, we expect different interactions (i.e. (i) both surgeons are from the intervention group, (ii) both surgeons are from the control group, and (iii) one surgeon is from the intervention group and the other from the control group). These different interactions were taken into account in the sample size calculation and will also be taken into account in the data analysis and allow for interesting future analyses. 

If a surgeon from the intervention group collaborates with a control group surgeon, we define that during the collaborative phase, the surgeon with a higher hierarchical status acts according to his or her study condition. Thus, an intervention group surgeon with lower hierarchical status will not have to initiate StOP?s in the presence of a control-group surgeon with a higher hierarchical status.

#### Steering committee

The entire study is coordinated by a steering committee. The steering committee consists of

- ○ Dr. Sandra Keller, post-doctoral fellow, work psychologist, project manager

The project manager will be responsible for organizing the information about the StOP?-protocol at the participating hospitals; for overseeing the recruitment of the surgeons on-site; for collecting the data related to the participating surgeons and hospitals; for organizing the StOP? training—including individual instruction-interviews with the surgeons in the interventional group; and for the implementation of the StOP?-protocol—including on-site observations and monthly feedbacks to each surgeon in the interventional group.

- ○ Prof. Guido Beldi, principal investigator, staff surgeon, Department for Visceral Surgery and Medicine, University of Bern, Switzerland

The principal investigator is responsible for the management and integrity of the design, conduct, and reporting of the research project and for managing, monitoring, and ensuring the integrity of any collaborative relationships. The PI ensures contact to the participating centers and ensures inclusion of sufficient participating centers.

- ○ Prof. Tanja Manser, co-principal investigator, Director School of Applied Psychology, University of Applied Sciences and Arts Northwestern Switzerland FHNW

The co-PI is responsible for the management and integrity of the design, conduct, and reporting of the research project and for managing, monitoring, and ensuring the integrity of any collaborative relationships. The co-PI ensures the scientific integrity of questions related to work psychology.

- ○ PD Dr. med. Sven Trelle, director, Clinical Trial Unit (CTU) Bern, University of Bern

Coordinates the study team of the CTU Bern and guarantees the statistical and analytical integrity of the trial. The CTU will be responsible for collecting patient data and will collaborate closely with the team of psychologists with regard to the different tasks listed above. The CTU is responsible for data analyses.

- ○ Prof. em. Franziska Tschan, professor emeritus for work psychology, University of Neuchâtel
- ○ Prof. em. Norbert Semmer, professor emeritus for work psychology, University of Bern

Prof. Tschan and Prof. Semmer were deeply involved in the StOP? I study as well as in the design of the current study and thereby add to the psychological expertise on communication and coordination in surgical teams, and they assure continuity between the first project and the current one.

The steering committee meets at least biweekly via videoconferencing. An agenda is sent at least a day beforehand and a protocol is written and distributed for each meeting by an administrator (Franka Theile). The steering committee is also responsible for the study in the coordinating center.

#### Advisory board

An advisory board has been established including representatives of patients, public, patient organizations, and medical faculties. Throughout the study, the advisory board shares findings of important research and raises the awareness among the public that the study is taking place. At later stages, the advisory board will analyze and comment on research findings from a patient perspective and support the dissemination of the findings. A meeting via videoconferencing is performed at least twice per year.

The advisory board consists of the following:

- *Representing patient safety*
  - ○ Prof. David Schwappach (Director Swiss Patient Safety Foundation, Switzerland, and Professor for Patient Safety at Institute of Social and Preventive Medicine, University of Bern)
  - ○ Prof. Matthias Weigl (Professor for Patient Safety, University Hospital Bonn, Germany; currently: Institute and Outpatient Clinic for Occupational, Social, and Environmental Medicine, LMU Munich, Germany)
  - ○ Prof. Siri Wiig, PhD (Center Director and Professor of Quality and Safety in Healthcare Systems, Center for Resilience in Healthcare, University of Stavanger, Norway)
- *Representing patients and the public*
  - ○ Prof. Stefan Weber (former dean of the ARTORG Institute, University of Bern, currently CEO CAScination AG)
- *Representing academic surgical units*
  - ○ PD Dr. Marco Von Strauss (staff surgeon, Clarunis, Department for abdominal surgery, University of Basel)
  - ○ Prof. Martin Hübner (professeur associé, Senior staff surgeon, Centre Hospitalier Universitaire Vaudois)

## Discussion

### Interventions that aim at changing behavior are notoriously difficult to implement

Problems of adherence to a protocol have been reported for the introduction of checklists [21] as well as for the implementation of training [26]. Often, such interventions are perceived as additional load (e.g., by interrupting routines), requiring additional time and implying behaviors perceived as awkward (e.g., introducing oneself to a familiar team). Adherence may be difficult to achieve even if participants acknowledge the usefulness of the procedure in principle but get frustrated by a suboptimal implementation process [47].

### The StOP?-protocol is no exception

We invested quite some effort in ensuring adherence during the previous study [31]. This included information to all team members and interviews with most surgeons above the resident level to ensure their collaboration. We also collected adherence data after each operation during the intervention period and provided monthly feedback about adherence. In addition, the StOP?-protocol was strongly supported by all department heads of the participating hospitals. All this resulted in an adherence rate of approximately 60%. Thus, there is room for improvement, although it should be added that our adherence rate was similar to those at the beginning of the WHO checklist introduction [48]; higher rates may be achieved only after an intervention has become widely implemented and accepted, as now seems to be the case for the surgical checklist [48]. Efforts in ensuring adherence will be a major focus of the project.

### Threat of bias of contamination

Given that the study aims to change the behavior of surgeons in the treatment group, potential bias or contamination of the surgeons enrolled in the control group needs to be addressed: First, although surgeons in the control group do not receive training, they will not be blind to the intervention, as surgeons within a department work closely together. This could be a source of contamination, because surgeons in the control group may observe StOP?s performed while they operate with a surgeon in the intervention group. If control group surgeons observe communication behavior that they judge as promising, they may be motivated to copy this behavior. However, sporadic observations of others’ behaviors are not sufficient to sustainably change a behavior. Training and feedbacks would be necessary for sustainable behavioral changes.

Second, some participating hospitals may have implemented a sign-out, which partially overlaps with a StOP?. For surgeons in the intervention group, the training interview will address this and ask the surgeons to reflect to what extent a StOP? may be associated to the sign-out, so that both can be combined during the operation.

## Trial status

The protocol version V1.2, 4.2.2022 was approved by the Lead Ethics Committee 14.3.2022. Recruitment of surgeons in the first participating center is planned for August 2022. Date of recruitment completed: October 2025.

## Dissemination of study results

The results of this study will be important for four key audiences, namely:

A: Healthcare professionals
B: Academia
C: Patients and the public
D: Policy makers and medical societies

If the study shows the expected results, the findings are of high importance and will be broadly received. Research is most effectively disseminated using multiple channels, adapted to the audiences.

During the study, we will build and maintain a network between participating study centers in order to exchange information, but also to provide regular feedback on the progress of the study. This will mostly be done using electronic means, but also regular videoconferencing and meetings. This network will also be used for the discussion and interpretation of the results (audiences A, B).

A webpage will be set up that allows communication with the study centers but also allows regular dissemination of information to an interested audience of healthcare professionals, media, and lay people (A, B, C, D). Via this webpage, webinars will be performed at least annually (A, B, C, D).

The results of the study will be published in peer-reviewed medical scientific journals and presented at national and international conferences (A, B, D). Newsletters will be sent by email (A, B, C, D). Mailing lists will be made available by the participating surgical societies and the two included patient organizations. After the study, toolkits of training materials will be offered free of charge (A). We will work closely with the media to inform the general public about the results of the study (A, B, C, D). Differences in outcome dependent on the gender of the surgeon as well as of the patient will be analyzed. The results of this analysis will be published (A, B, C, D)

## Reproducibility

We will deposit the final protocol in a preprint repository (medRxiv.org) and submit it to a peer-reviewed journal. At publication of the primary results, the full protocol, the data management plan including the codebook of the trial database, and information on how to get access to the primary trial data will be published in the repository of the University of Bern which fulfills the FAIR criteria (Bern Open Repository and Information System (boris.unibe.ch)). A 12-month grace period after publication of the primary results is currently foreseen to allow all collaborators to complete additional, pre-planned analyses and projects.

## Supplementary Information

**Additional file 1.**

## Data Availability

Data will be deposited in the Bern Open Repository and Information System Research Data (BORIS-RD). BORIS-RD allows searching and is indexed by search engines. All items are stored with a unique Digital Object Identifier (DOI) that can be referenced in respective publications. It should be noted that we plan to register and deposit data that relates to individual, primary publications and not the whole study database as such. This enables other researchers to replicate published analyses and results as well as to re-use the data for additional analyses such as meta-analysis.

## Abbreviations

CI: Confidence interval
CONSORT: Consolidated Standards of Reporting Trials
ICC: Intracluster correlation coefficient
ITT: Intention-to-treat
SSI: Surgical site infection
WHO: World Health Organization
