# Supplementary material for: StOP? II trial: cluster randomized clinical trial to test the implementation of a toolbox for structured communication in the operating room—study protocol
Source: Trials. 2022 Oct 18;23:878. doi: 10.1186/s13063-022-06775-y (PMC9580155; doi:10.1186/s13063-022-06775-y)
Supplement: Supplementary file 1 — Additional file 1. [file 13063_2022_6775_MOESM1_ESM.pdf]

Hospital data

Record ID ________________________________

Name of hospital ________________________________

Hospital ID ________________________________

Full Address of the hospital ________________________________

Country of hospital ________________________________

(EJ: I'd like to implement a dropdown incl. options instead of offering a text field.)

Language region of Hospital ________________________________

(EJ: I'd like to implement a dropdown incl. options instead of offering a text field.)

Type of Hospital ________________________________

(EJ: I'd like to implement a dropdown incl. options instead of offering a text field.)

Number of beds of the hospital ________________________________
(ranges to be defined)

Teaching category of hospital ________________________________

(EJ: I'd like to implement a dropdown incl. options instead of offering a text field.)

Number of employees within the hospital ________________________________
(ranges to be defined)

Number of operation rooms in the hospital without rooms exclusively for outpatient procedures ________________________________
(ranges to be defined)

Unit Data

Record ID	__________________________________
Unit name	__________________________________
Main contact person within unit	__________________________________
Annual number of operations without outpatient procedures	__________________________________ (ranges to be defined)
Number of attending surgeons employed by the hospital within the study unit	__________________________________ (ranges to be defined)

Protocol Deviation

Record ID	______________________
Protocol deviation	◯ Yes<br>◯ No
Date of protocol deviation awareness	______________________<br>(DD.MM.YYYY, ranges to be defined)
Operation ID	______________________
Deviation category	◯ Eligibility / Enrollment<br>◯ Informed consent<br>◯ Randomization / Treatment allocation<br>◯ Blinding procedure<br>◯ Withdrawal criteria<br>◯ Treatment compliance<br>◯ Primary endpoint assessment<br>◯ Missed assessment<br>◯ Other
If other, specify	______________________
Did the surgeon receive a wrong information about his group assignment?	◯ Yes<br>◯ No
Please enter communicated group assignment	◯ StOP? ◯ Control group
Brief description of deviation	______________________

Surgeon (Research Team)

Record ID

____

Hospital ID

◯ Inselspital
◯ CHUV
◯ Hospital A
◯ Hospital B
◯ Insel
◯ Regionalspital THun
◯ RST
◯ Spital Burgdorf
(If hospital is not yet listed, please add it here: https://redcap.ctu.unibe.ch/redcap_v11.1.10/index.php?pid=1088)

Unit ID

Surgeon ID

____

Inclusion Criteria for Surgeons

Does the surgeon have a specialization in one of the following disciplines:

general, visceral, thoracic, vascular surgery, surgical urology or gynecology

◯ Yes
◯ No

Is the surgeon board-certified

◯ Yes
◯ No

Exclusion Criteria for Surgeons

Is the surgeon already performing StOP?s

◯ Yes
◯ No

Was the surgeon previously enrolled in the trial

◯ Yes
◯ No

Surgeon is ELIGIBLE

Patient does NOT meet eligibility criteria

Group assignment

Group allocation / Randomization

◯ Control Group
◯ StOP? Group

Time period the surgeon participates in the study

(EJ: how should this field been filled in?)

____

Training and Retraining

Training: Received the training material (documents)

◯ Yes
◯ No

Date of interview

____
(DD.MM.YYYY, ranges to be defined)

When was the surgeon observed and received a feedback on the StOP?s performed immediately after operations

____
(DD.MM.YYYY, ranges to be defined)

When did the surgeon receive feedback on adherence

____
(DD.MM.YYYY, ranges to be defined)

Was retraining done with this surgeon because of adherence problems?

◯ Yes
◯ No

When was the surgeon retrained?

____
(DD.MM.YYYY, ranges to be defined)

Remarks with regard to the surgeon

____

Surgeon Survey

Please complete the survey below.

Thank you!

What are your professional titles?	☐ Professor<br>☐ PD<br>☐ MD<br>☐ PhD
What is your first name?	______________________
What is your last name?	______________________
What is your date of birth?	______________________<br>(DD.MM.YYYY, ranges to be defined)
Gender	◯ Male<br>◯ Female
Nationality/Nationalities	☐ Switzerland<br>☐ Germany<br>☐ France<br>☐ Italy<br>☐ Austria<br>☐ Other
Please specify	______________________
What are your native languages?	☐ French<br>☐ German<br>☐ Italian<br>☐ Other
Please specify	______________________
When did you obtain your general surgery title?	______________________<br>(DD.MM.YYYY, ranges to be defined)
When did you obtain your surgical specialization title?	______________________<br>(DD.MM.YYYY, ranges to be defined)

What are your specialized degrees?

- ◯ Allergologie und klinische Immunologie/Allergologie et immunologie clinique
- ◯ Allgemeine Innere Medizin/ Médecine interne générale
- ◯ Anästhesiologie/Anesthésiologie
- ◯ Angiologie/Angiologie
- ◯ Arbeitsmedizin/Médecine du travail
- ◯ Chirurgie/Chirurgie
- ◯ Dermatologie und Venerologie/Dermatologie et vénéréologie
- ◯ Endokrinologie und Diabetologie/Endocrinologie et diabétologie
- ◯ Gastroenterologie/Gastroentérologie
- ◯ Gefässchirurgie/Chirurgie vasculaire
- ◯ Gynäkologie und Geburtshilfe/Gynécologie et obstétrique
- ◯ Hämatologie/Hématologie
- ◯ Handchirurgie/Chirurgie de la main
- ◯ Herz- und thorakale Gefässchirurgie/Chirurgie cardiaque et vasculaire thoracique
- ◯ Infektiologie/Infectiologie
- ◯ Intensivmedizin/Médecine intensive
- ◯ Kardiologie/Cardiologie
- ◯ Kinder- und Jugendmedizin/Pédiatrie
- ◯ Kinder- und Jugendpsychiatrie und -psychotherapie/Psychiatrie et psychothérapie d'enfants et d'adolescents
- ◯ Kinderchirurgie/Chirurgie pédiatrique
- ◯ Klinische Pharmakologie und Toxikologie/Pharmacologie et toxicologie cliniques
- ◯ Medizinische Genetik/Génétique médicale
- ◯ Medizinische Onkologie/Oncologie médicale
- ◯ Kiefer- und Gesichtschirurgie/Chirurgie orale et maxillo-faciale
- ◯ Nephrologie/Néphrologie
- ◯ Neurochirurgie/Neurochirurgie
- ◯ Neurologie/Neurologie
- ◯ Nuklearmedizin/Médecine nucléaire
- ◯ Ophthalmologie/Ophthalmologie
- ◯ Orthopädische Chirurgie und Traumatologie des Bewegungsapparates/Chirurgie orthopédique et traumatologie de l'appareil locomoteur
- ◯ Oto-Rhino-Laryngologie/Oto-Rhino-Laryngologie
- ◯ Pathologie/Pathologie
- ◯ Pharmazeutische Medizin/Médecine pharmaceutique
- ◯ Physikalische Medizin und Rehabilitation/Médecine physique et réadaptation
- ◯ Rekonstruktive und Ästhetische Chirurgie/Chirurgie plastique, reconstructive et esthétique
- ◯ Pneumologie/Pneumologie
- ◯ Prävention und Public Health/Prévention et santé publique
- ◯ Psychiatrie und Psychotherapie/Psychiatrie et psychothérapie
- ◯ Radiologie/Radiologie
- ◯ Radio-Onkologie und Strahlentherapie/Radio-oncologie et radiothérapie
- ◯ Rechtsmedizin/Médecine légale
- ◯ Rheumatologie/Rhumatologie
- ◯ Thoraxchirurgie/Chirurgie thoracique
- ◯ Tropen- und Reisemedizin/Médecine tropicale et médecine des voyages
- ◯ Urologie/Urologie

When did you first start working at your current hospital?	____________________ (DD.MM.YYYY, ranges to be defined)
How many months have you been working at your current hospital?<br><br>(EJ: this question is redundant)	____________________
What is your current position?	◯ Leitender Arzt/ Médecin adjoint<br>◯ Oberarzt /Chef de clinique<br>◯ Stv. Oberarzt /Chef de clinique adjoint
Contact e-mail address	____________________

Compliance with StOP? / Scrub Technician Questionnaire (Psychologists)

Record ID	__________________________________
SK: For the last question and the rest: are checkboxes or drop down lists better?	
Questionnaire on compliance available	◯ Yes<br>◯ No
Date	__________________________________<br>(Operation date, as on questionnaire)
Presence of scrub	◯ Complete operation<br>◯ Part of the operation
Present from ...	__________________________________<br>(HH:MM, ranges to be defined)
Present to ...	__________________________________<br>(HH:MM, ranges to be defined)
Was the "StOP?" annouced at timeout?	◯ Ja<br>◯ Nein<br>◯ War nicht anwesend beim Timeout<br>◯ Es wurde kein Timeout gemacht
How good was the timeout?<br><br>Good StOP?:<br>aloud, complete (status, objectives, problems, questions), dedicated, the whole team paused and paid attention	◯ Very bad<br>◯ Rather bad<br>◯ Medium<br>◯ Rather good<br>◯ Very good
How many StOP?s were carried out?	__________________________________<br>(during the time the scrub technician was present)
How good was/were the "StOP?"s carried out?	◯ Very bad<br>◯ Rather bad<br>◯ Medium<br>◯ Rather good<br>◯ Very good

Please complete for all operations:

How good was the collaboration during the surgery?	◯ Very bad<br>◯ Bad<br>◯ Medium<br>◯ Good<br>◯ Very good
Was there a restless, hectic atmosphere in the operating room?	◯ very calm<br>◯ calm<br>◯ medium<br>◯ hectic<br>◯ very hectic
Was there any tension or conflict during the surgery?	◯ none<br>◯ little<br>◯ few<br>◯ many<br>◯ very many
Were there any critical situations for the patient during surgery?	◯ Yes<br>◯ No
Please describe the critical situation(s) here in keywords	____
Comments / Notes	____

Basic Information on Operation (Psychologist)

Record ID __________________________________

ID Assignment

Operation ID

Please click on "Randomize" in order to assign a unique Operation ID:

◯ OP-83604373
◯ OP-98892836
◯ OP-79717517
◯ OP-49486021
◯ OP-14719557
◯ OP-20867309
◯ OP-37844720
◯ OP-10830775
◯ OP-57511189
◯ OP-96779852
◯ OP-53642718
◯ OP-56437873
◯ OP-65033572
◯ OP-45176187
◯ OP-60573764
◯ OP-79188983
◯ OP-63200847
◯ OP-59143208
◯ OP-53830275
◯ OP-85528572
◯ OP-41607196
◯ OP-90536368
◯ OP-71746149
◯ OP-67078060
◯ OP-15258741
◯ OP-48082779
◯ OP-13092236
◯ OP-22673262
◯ OP-40117663
◯ OP-15644794
◯ OP-88132603
◯ OP-95344918
◯ OP-20796898
◯ OP-38497663
◯ OP-53897857
◯ OP-98102457
◯ OP-32380713
◯ OP-32889431
◯ OP-44248237
◯ OP-15795073
◯ OP-91115547
◯ OP-76577363
◯ OP-28148683
◯ OP-76922335
◯ OP-42054227
◯ OP-67453467
◯ OP-95702603
◯ OP-34738521
◯ OP-59631511
◯ OP-84146339
◯ OP-44723538
◯ OP-41346661
◯ OP-37553179
◯ OP-83925352
◯ OP-32464580
◯ OP-70733725
◯ OP-45451339
◯ OP-40543034
◯ OP-57781353
◯ OP-67088760
◯ OP-64328183
◯ OP-92095315
◯ OP-50916955
◯ OP-36483443
◯ OP-24847958
◯ OP-55520843
◯ OP-48418469
◯ OP-68681825
◯ OP-73654351

◯ OP-86912599
◯ OP-12480530
◯ OP-48948377
◯ OP-37905875
◯ OP-66164471
◯ OP-69296381
◯ OP-52428466
◯ OP-41349100
◯ OP-61688868
◯ OP-23130857
◯ OP-18627597
◯ OP-52480583
◯ OP-66576807
◯ OP-16518291
◯ OP-82069572
◯ OP-70352554
◯ OP-82092651
◯ OP-27103400
◯ OP-14477681
◯ OP-95404396
◯ OP-14241457
◯ OP-41045031
◯ OP-36110055
◯ OP-98552710
◯ OP-62263528
◯ OP-76606338
◯ OP-42898941
◯ OP-14930415
◯ OP-60359555
◯ OP-10938548
◯ OP-48933442
◯ OP-29916382
◯ OP-70158126
◯ OP-29696438
◯ OP-90842956
◯ OP-84391057
◯ OP-88976245
◯ OP-20109946
◯ OP-83160341
◯ OP-94818735
◯ OP-56146996
◯ OP-11732595
◯ OP-18416875
◯ OP-39020026
◯ OP-52243963
◯ OP-56460796
◯ OP-83060810
◯ OP-14893996
◯ OP-68591034
◯ OP-76630360
◯ OP-18970588
◯ OP-52777959
◯ OP-27404057
◯ OP-97690555
◯ OP-51033264
◯ OP-89144198
◯ OP-84280566
◯ OP-11186842
◯ OP-11592060
◯ OP-81366622
◯ OP-17354822
◯ OP-80717000
◯ OP-95139879
◯ OP-75968454
◯ OP-87254379
◯ OP-91222364
◯ OP-88234593
◯ OP-23034070
◯ OP-78378945
◯ OP-13463545
◯ OP-96578460

◯ OP-22832272
◯ OP-78549701
◯ OP-49282725
◯ OP-78775755
◯ OP-36107849
◯ OP-82805631
◯ OP-90829669
◯ OP-14440661
◯ OP-26266429
◯ OP-34233044
◯ OP-77079970
◯ OP-68982830
◯ OP-44057539
◯ OP-65674319
◯ OP-50136169
◯ OP-51367722
◯ OP-40028530
◯ OP-59156990
◯ OP-54286575
◯ OP-23267848
◯ OP-26252805
◯ OP-17089866
◯ OP-18052191
◯ OP-24672600
◯ OP-42092580
◯ OP-76856998
◯ OP-73478793
◯ OP-78069613
◯ OP-63257301
◯ OP-28016989
◯ OP-11073365
◯ OP-72038940
◯ OP-87822981
◯ OP-19501123
◯ OP-89270570
◯ OP-56051630
◯ OP-88266615
◯ OP-47683403
◯ OP-96429307
◯ OP-63651462
◯ OP-42544022
◯ OP-51583381
◯ OP-99461687
◯ OP-93579513
◯ OP-78092465
◯ OP-56897665
◯ OP-88659324
◯ OP-77138817
◯ OP-89034693
◯ OP-15096669
◯ OP-51430756
◯ OP-52906019
◯ OP-70972611
◯ OP-26683813
◯ OP-47205494
◯ OP-83183132
◯ OP-16752304
◯ OP-96277533
◯ OP-30429812
◯ OP-59941506
◯ OP-58452630
◯ OP-74284443
◯ OP-29661462
◯ OP-65880830
◯ OP-66674449
◯ OP-56287340
◯ OP-76054702
◯ OP-86533317
◯ OP-44330289
◯ OP-99045259
◯ OP-96460138

◯ OP-83299536
◯ OP-59100760
◯ OP-56398613
◯ OP-24647834
◯ OP-65509591
◯ OP-96223731
◯ OP-29248691
◯ OP-33785903
◯ OP-86356197
◯ OP-11542543
◯ OP-83276937
◯ OP-61704289
◯ OP-19082014
◯ OP-36664318
◯ OP-38341191
◯ OP-34337582
◯ OP-91065167
◯ OP-18137332
◯ OP-78201337
◯ OP-77680774
◯ OP-81299228
◯ OP-41257195
◯ OP-65270071
◯ OP-94203428
◯ OP-67464815
◯ OP-43419318
◯ OP-29161792
◯ OP-71387635
◯ OP-99738504
◯ OP-46958323
◯ OP-76142151

Operation ID ______________________________

VERY IMPORTANT

PLEASE SAVE this form and then COPY the Operation ID into the REDCap data base with the patient identifying information and fill up the patient identifiable information before continuing here!!

Link to the respective data base:

StOP?: Patient Identifying Information.

Operation ID copy pasted into data base "StOP?: Patient Identifying Information"?

◯ Yes
◯ No

Please copy paste the patient's calculated age from the Patient Identifying Information Data Base

____
(age in years, 2 decimals after comma)

Link to the respective data base:

StOP?: Patient Identifying Information.

Inclusion/Exclusion of Operation

Was the procedures done in an outpatient clinics, on wards, etc.?

◯ Yes
◯ No

EJ: these two criteria are listed as inclusion/exclusion criteria in the protocol.
Where should we place them and how should they be rephrased (needs to be a yes/no question)

Mainly diagnostic endoscopic procedures (e.g. colonoscopy, gastroscopy, bronchoscopy)

Percutaenous interventions (e.g., transurethral interventions)

◯ Yes
◯ No

Hospital and Unit

Hospital (SQL)

◯ Inselspital
◯ CHUV
◯ Hospital A
◯ Hospital B
◯ Insel
◯ Regionalspital THun
◯ RST
◯ Spital Burgdorf
(If hospital is not yet listed, please add it here: StOP?: Hospitals and Units)

Unit (SQL)

Comment: after discussing this, the current plan is to provide a structured list instead of the SQL drop-down (issue with SQL: form needs to be saved after hospital selection in order to allow the filtering of the applicable units for the selected hospital)

Suggestion=> since OP-ID cannot be copied as long the form is not saved, I suggest to move the hospital selection field to the top as well and instruct the data entry personal to 1. select the hospital, 2. randomize, 3. save the form, 4. copy the record and OP-ID into the identifying database.

Operation

Type of operation

____
(copy text exactly from file (operation list))

Date of operation

____
(DD.MM.YYYY; ranges to be defined)

Start of operation

____
(HH:MM, start = incision access site(s), ranges to be defined)

End of operation	__________________________________<br>(HH:MM, stop = closure access site(s), ranges to be defined)
Main Surgeon	◯ Doctor Lovejoy<br>◯ Doctor Strange<br>◯ Dr F. Bader<br>◯ Dr L. Amsler<br>◯ Dr. Heiri<br>◯ Peter Test<br>◯ Prof G. Gerber<br>◯ Test Peter
Main surgeon's randomization group assignment:	◯ StOP? Surgeon<br>◯ Control Group Surgeon
Second Surgeon	◯ Doctor Lovejoy<br>◯ Doctor Strange<br>◯ Dr F. Bader<br>◯ Dr L. Amsler<br>◯ Dr. Heiri<br>◯ Peter Test<br>◯ Prof G. Gerber<br>◯ Test Peter
Second surgeon's randomization group assignment:	◯ StOP? Surgeon<br>◯ Control Group Surgeon

Clinical Data & Follow Up 30 Days (Study Nurses)

Record ID ________________

OPERATION NUMBER & TYPE:
[baseline_arm_1][pk_operation_id], [baseline_arm_1][op_type_txt]

Date and Starting Time of Main Operation:
[baseline_arm_1][bi_op_date], [baseline_arm_1][op_start_time]

Patient Characteristics

Gender
- ◯ Male
- ◯ Female
- ◯ Other

Patient age at operation (read-only):
[fk_pat_age] years

Patient height [cm] ________________
(format = integer; unit = cm; min = 120, max = 220)

Patient weight [kg] ________________
(format = 1 decimal place; unit = kg; min = 30.0, max = 150.0)

BMI (calculated) ________________

ASA score
- ◯ ASA 1
- ◯ ASA 2
- ◯ ASA 3
- ◯ ASA 4
- ◯ ASA 5
- ◯ Missing

Hospital Stay and Discharge (Main Operation)

Date of hospital admission ________________
(DD.MM.YYYY, ranges to be defined)

Hospitalization ongoing?
- ◯ Yes
- ◯ No
- ◯ 'Date of discharge' missing forever

Date of hospital discharge ________________
(DD.MM.YYYY, ranges to be defined)

Length of hospital stay [days, calculated] ________________

Operation Team

Main surgeon	◯ Doctor Lovejoy<br>◯ Doctor Strange<br>◯ Dr F. Bader<br>◯ Dr L. Amsler<br>◯ Dr. Heiri<br>◯ Peter Test<br>◯ Prof G. Gerber<br>◯ Test Peter
Main surgeon not on the list:	______________________
Second surgeon present?	◯ Yes<br>◯ No
Second surgeon	◯ Doctor Lovejoy<br>◯ Doctor Strange<br>◯ Dr F. Bader<br>◯ Dr L. Amsler<br>◯ Dr. Heiri<br>◯ Peter Test<br>◯ Prof G. Gerber<br>◯ Test Peter
Second surgeon not on the list:	______________________
Resident surgeon 1 present?	◯ Yes<br>◯ No
Resident surgeon 1	______________________
Resident surgeon 2 present?	◯ Yes<br>◯ No
Resident surgeon 2	______________________
Resident surgeon 2 present?	◯ Yes<br>◯ No
Resident surgeon 3	______________________
Other surgeon(s) present?	◯ Yes<br>◯ No
Other surgeons present	______________________

Operation

Type of main operation (coded)	◯ Type of operation unclear (please specify)<br>◯ AAA - Abdominal aortic aneurysm repair<br>◯ HYST - Abdominal hysterectomy<br>◯ AMP - Limb amputation<br>◯ APPY - Appendix surgery<br>◯ AVSD - AV shunt for dialysis<br>◯ BILIB - Bile duct operation<br>◯ BILIL - Liver surgery<br>◯ LTP - Liver transplant<br>◯ BILIP - Pancreas surgery<br>◯ BRST - Breast surgery<br>◯ CEA - Carotid endarterectomy<br>◯ CHOL - Gallbladder surgery<br>◯ COLO - Colon surgery<br>◯ CSEC - Cesarean section<br>◯ FX - Open reduction of fracture<br>◯ GAST - Gastric surgery<br>◯ HER - Herniorrhaphy<br>◯ NEPH - Kidney surgery<br>◯ KTP - Kidney transplant<br>◯ OVRY - Ovarian surgery<br>◯ PRST - Prostate surgery<br>◯ REC - Rectal surgery<br>◯ SB - Small bowel surgery<br>◯ SPLE - Spleen surgery<br>◯ THOR - Thoracic surgery<br>◯ THYR - Thyroid and/or parathyroid surgery<br>◯ VHYS - Vaginal hysterectomy<br>◯ VSHN - Ventricular shunt<br>◯ XLAP - Exploratory laparotomy
Please copy paste available information about type of operation into this text box:	______________________________
Did the patient had a second operation(s) (besides of the index operation)?	◯ Yes<br>◯ No
How many additional operations?	◯ 1<br>◯ 2<br>◯ 3

Type of additional operation 1

- ◯ AAA - Abdominal aortic aneurysm repair
- ◯ AMP - Limb amputation
- ◯ APPY - Appendix surgery
- ◯ AVSD - AV shunt for dialysis
- ◯ BILIB - Bile duct operation
- ◯ BILIL - Liver surgery
- ◯ BILIP - Pancreas surgery
- ◯ BRST - Breast surgery
- ◯ CEA - Carotid endarterectomy
- ◯ CHOL - Gallbladder surgery
- ◯ COLO - Colon surgery
- ◯ CSEC - Cesarean section
- ◯ FX - Open reduction of fracture
- ◯ GAST - Gastric surgery
- ◯ HER - Herniorrhaphy
- ◯ HYST - Abdominal hysterectomy
- ◯ KTP - Kidney transplant
- ◯ LTP - Liver transplant
- ◯ NEPH - Kidney surgery
- ◯ OVRY - Ovarian surgery
- ◯ PRST - Prostate surgery
- ◯ REC - Rectal surgery
- ◯ SB - Small bowel surgery
- ◯ SPLE - Spleen surgery
- ◯ THOR - Thoracic surgery
- ◯ THYR - Thyroid and/or parathyroid surgery
- ◯ VHYS - Vaginal hysterectomy
- ◯ VSHN - Ventricular shunt
- ◯ XLAP - Exploratory laparotomy

Type of additional operation 2

- ◯ AAA - Abdominal aortic aneurysm repair
- ◯ AMP - Limb amputation
- ◯ APPY - Appendix surgery
- ◯ AVSD - AV shunt for dialysis
- ◯ BILIB - Bile duct operation
- ◯ BILIL - Liver surgery
- ◯ BILIP - Pancreas surgery
- ◯ BRST - Breast surgery
- ◯ CEA - Carotid endarterectomy
- ◯ CHOL - Gallbladder surgery
- ◯ COLO - Colon surgery
- ◯ CSEC - Cesarean section
- ◯ FX - Open reduction of fracture
- ◯ GAST - Gastric surgery
- ◯ HER - Herniorrhaphy
- ◯ HYST - Abdominal hysterectomy
- ◯ KTP - Kidney transplant
- ◯ LTP - Liver transplant
- ◯ NEPH - Kidney surgery
- ◯ OVRY - Ovarian surgery
- ◯ PRST - Prostate surgery
- ◯ REC - Rectal surgery
- ◯ SB - Small bowel surgery
- ◯ SPLE - Spleen surgery
- ◯ THOR - Thoracic surgery
- ◯ THYR - Thyroid and/or parathyroid surgery
- ◯ VHYS - Vaginal hysterectomy
- ◯ VSHN - Ventricular shunt
- ◯ XLAP - Exploratory laparotomy

Type of additional operation 3	◯ AAA - Abdominal aortic aneurysm repair<br>◯ AMP - Limb amputation<br>◯ APPY - Appendix surgery<br>◯ AVSD - AV shunt for dialysis<br>◯ BILIB - Bile duct operation<br>◯ BILIL - Liver surgery<br>◯ BILIP - Pancreas surgery<br>◯ BRST - Breast surgery<br>◯ CEA - Carotid endarterectomy<br>◯ CHOL - Gallbladder surgery<br>◯ COLO - Colon surgery<br>◯ CSEC - Cesarean section<br>◯ FX - Open reduction of fracture<br>◯ GAST - Gastric surgery<br>◯ HER - Herniorrhaphy<br>◯ HYST - Abdominal hysterectomy<br>◯ KTP - Kidney transplant<br>◯ LTP - Liver transplant<br>◯ NEPH - Kidney surgery<br>◯ OVRY - Ovarian surgery<br>◯ PRST - Prostate surgery<br>◯ REC - Rectal surgery<br>◯ SB - Small bowel surgery<br>◯ SPLE - Spleen surgery<br>◯ THOR - Thoracic surgery<br>◯ THYR - Thyroid and/or parathyroid surgery<br>◯ VHYS - Vaginal hysterectomy<br>◯ VSHN - Ventricular shunt<br>◯ XLAP - Exploratory laparotomy
Surgical Access main operation	◯ open<br>◯ minimal invasive<br>◯ converted<br>◯ robotic
Emergency operation?	◯ Yes<br>◯ No
Wound contamination	◯ clean<br>◯ clean-contaminated<br>◯ contaminated<br>◯ dirty<br>◯ Wound contamination status unclear (please specify)
Description of problem with wound contamination calssification	______________________________
Was there a laparotomy on the same site between [fup_laparotomy_calcdate] (= date of operation minus 30 days) and [baseline_arm_1][bi_op_date] (= date of operation)?	◯ Yes<br>◯ No
=> Relevant date: operation date minus 30 days	________________________
Date of laparotomy	________________________<br>(DD.MM.YYYY, ranges to be defined)

Re-Operations, Re-Interventions, Complications

Unplanned re-operation or re-intervention under general anesthesia between [baseline_arm_1][bi_op_date] (= date of operation) and [fup_reop_reinterv_calcdate] (= date of operation plus 30 days)?	○ Yes<br>○ No
=> Relevant date: operation date plus 30 days	______________________
Date of reoperation or reintervention under general anesthesia	______________________<br>(enter the date of the first re-operation or re-intervention, DD.MM.YYYY, ranges to be defined)
Reason for reoperation or reintervention under general anesthesia	____________________________
Complication requiring ICU management (not part of the initial care plan and excluding ICU stay immediately after operation)	○ Yes<br>○ No<br>(within 30 days)
Classification of surgical-induced complication<br><br>(NOT the admission indication, but due to the surgery)<br><br>@Dik => To be discussed with study team if really needed and if this is really available for the study nurses	○ I - Any deviation from the normal postoperative course without the need for pharmacological treatment or surgical, endoscopic and radiological interventions allowed therapeutic regimens are: drugs as antiemetics, antipyretics, analgetics, diuretics and electrolytes and physiotherapy. This grade also includes wound infections opened at the bedside.<br>○ II - Requiring pharmacological treatment with drugs other than such allowed for grade I complications. Blood transfusionsand total parenteral nutritionare also included.<br>○ IIIa - Requiring surgical, endoscopic or radiological intervention: Intervention not under general anesthesia<br>○ IIIb - Requiring surgical, endoscopic or radiological intervention: Intervention under general anesthesia<br>○ IVa - Life-threatening complication (including CNS complications)* requiring IC/ICU-management with single organ dysfunction (including dialysis)<br>○ IVb - Life-threatening complication (including CNS complications)* requiring IC/ICU-management with multiorgandysfunction<br>○ V - Death of a patient
Date of complication requiring ICU management	______________________<br>(within 30 days after operation, DD.MM.YYYY, ranges to be defined)
Reason of complication requiring ICU management	____________________________<br>(within 30 days after operation)

Birth defects of babies born within 30 days after the operation?<br><br>Comment: This item needs to be re-discussed by the study team	◯ Yes<br>◯ No<br>(within 30 days after operation)
Reason for birth defects of babies born within 30 days after the operation	__________________<br>(within 30 days after operation)

Unplanned Hospital Readmission

Unplanned readmission between [baseline_arm_1][bi_op_date] (= date of operation) and [fup_unpl_readm_calcdate] (= date of operation plus 30 days)?	◯ Yes<br>◯ No
=> Relevant date: operation date plus 30 days	__________________
Date of readmission	__________________<br>(within 30 days after operation, DD.MM.YYYY, ranges to be defined)
Date of discharge after readmission	__________________<br>(within 30 days after operation, DD.MM.YYYY, ranges to be defined)
Reason for hospital readmission	__________________<br>(within 30 days after operation)

Serious Adverse Event

Any serious adverse event that may be associated to the StOP?-protocol between [baseline_arm_1][bi_op_date] (= date of operation) and [fup_sae_calcdate] (= date of operation plus 30 days)?	◯ Yes<br>◯ No
=> Relevant date: operation date plus 30 days	__________________
Date of serious adverse event potentially associated to the StOP?-protocol	__________________<br>(within 30 days after operation, DD.MM.YYYY, ranges to be defined)
Description of the serious adverse event potentially associated to the StOP?-protocol	__________________<br>(within 30 days after operation)

Patient Status

Patient alive after the following date?

____

◯ Yes
◯ No
◯ Unclear
◯ Mortality status missing forever

Date of exitus letalis

____
(DD.MM.YYYY, ranges to be defined)

Reason of exitus letalis

____

Notes & Comments

General remarks on the data of this patient

____

Patient Assessment: Eligible for Follow Up? (Study Nurses)

Record ID	__________________________________

Eligibility Assessment

Consent available?	◯ Yes - General Consent<br>◯ No<br>◯ No - inclusion based on HRA article 34
Patient age below 18 years?	◯ Yes<br>◯ No
Previous operation at the same site up to 30 days prior the index operation	◯ Yes<br>◯ No
Patient is ELIGIBLE	
Patient does NOT meet eligibility criteria	

Patient Information

Date of operation	__________________________________

Patient (Contact Information)

Record ID ______________________

Patient Identification Number (PID) ______________________

Patient Case Number (FID) ______________________

Please copy paste the corresponding Operation ID from the Clinical Data / Operations Data Base ______________________
(This is the Operation ID to be found in the Clinical Data / Operations Data Base)

Link to the respective data base:

StOP?: Clinical Data / Operations

First name ______________________

Family name ______________________

Date of birth ______________________
(DD.MM.YYYY, ranges to be defined)

Operation related data

Hospital
- ◯ Inselspital
- ◯ CHUV
- ◯ Hospital A
- ◯ Hospital B
- ◯ Insel
- ◯ Regionalspital THun
- ◯ RST
- ◯ Spital Burgdorf

Unit ______________________

Comment: after discussing this, the current plan is to provide a structured list instead of the SQL drop-down (issue with SQL: form needs to be saved after hospital selection in order to allow the filtering of the applicable units for the selected hospital)

Date of operation ______________________
(DD.MM.YYYY, ranges to be defined)

VERY IMPORTANT

PLEASE SAVE this form and then COPY the patient's calculated age into the REDCap data base with the basic clinical information!!

Link to the respective data base:

Age at operation (calculated)

____
(years (rounded to 2 decimals))

Patient's calculated age at operation copy pasted into data base "StOP?: Clinical Data / Operations"?

◯ Yes
◯ No
